# Systemic therapy for hepatocellular carcinoma: trial enrichment does not guarantee success

**DOI:** 10.18632/oncotarget.26052

**Published:** 2018-09-18

**Authors:** Leonardo G. Da Fonseca, Maria Reig, Jordi Bruix

**Affiliations:** Jordi Bruix: BCLC group, Liver Unit, Hospital Clínic de Barcelona, IDIBAPS, CIBERehd, Universitat de Barcelona, Barcelona, Spain

**Keywords:** hepatocellular carcinoma, outcome, systemic therapy, trial design, MET expression

The view of cancer as an anatomic-defined disease has shifted to a complex understanding at a cellular and molecular level, including the role of microenvironment. As a consequence, the focus is placed on the search for drugs that target cancer progression mechanisms. This implies the need to identify targetable predictive biomarkers and stratify patients that may benefit from a biomarker-driven approach. This has been achieved for many agents currently used in oncology, but in other cases it is not an easy task.

The advances obtained in systemic therapy for hepatocellular carcinoma (HCC) in the last decade were remarkable, although the number of negative clinical trials may prime an erroneous conclusion that little progress was reached. The sequential approach with sorafenib followed by regorafenib significantly extends survival beyond 2 years. At the same time, lenvatinib demonstrated to be non-inferior to sorafenib as first-line therapy. In addition, we have two additional effective agents after sorafenib failure and the decision on how to select the best treatment is becoming an important issue [1].

Biomarker driven treatment was addressed in the phase III METIV trial, which tested tivantinib in patients with high MET expression who were previously treated with sorafenib [2]. MET is the receptor for hepatocyte growth factor, that activates the MAPK and PI3K-AKT pathways in order to promote tumor development. MET expression was an adverse prognostic factor and MET is more frequently overexpressed in tumor tissue after sorafenib. A phase II trial showed that tivantinib improved survival in patients with MET-high [3]. Unfortunately, despite this background, the phase III trial did not confirm the impact in survival. The possible causes for these results include differences between the phases II and III designs: sample size, tivantinib formulation, different laboratories evaluating MET expression, number of biopsies obtained before and after sorafenib, number of patients with MET-high tumors and the protocol-specified requirement for biopsy results to be available before enrollment in the phase III. This may have selected patients who maintained a good performance status despite progression under sorafenib and time elapsed between trial consideration and randomization. This may result in a subgroup of patients with less-aggressive disease.

Discrepancies between results of phase II and phase III trials are not that unusual. The U.S. Food and Drug Administration published 22 case studies of drugs, vaccines and medical devices in which promising phase II results were not confirmed in phase III, even when the phase II was relatively large and assessed clinical outcomes [4]. This illustrates the importance of critically discussing the reasons of phase III failures in order to provide concepts for future trials in a specific disease.

HCC is a unique case not only because of the underlying liver cirrhosis, but also because there are no surrogate parameters for OS. Benefits of sorafenib occur in the absence of a significant rate of responses according to common radiology criteria and data from the pivotal trials discard time-to-progression on sorafenib as a valid surrogate [5]. Indeed, pattern of progression is more relevant in predicting outcome. Development of new extra-hepatic lesions carries a poorer prognosis as compared to other patterns. For these reasons, the results of phase III trials designed to test the impact of the immune-check points inhibitors on overall survival are eagerly awaited irrespective of their appealing response rate: around 20% for both nivolumab and pembrolizumab [1].

Regarding the results of METIV, should we abandon the search for predictive biomarkers in HCC, in particular MET? Probably not. Cabozantinib, a kinase-inhibitor directed to MET and vascular endothelial growth factor receptor 2 (VEGFR2), was shown to significantly improve the overall survival of HCC patients submitted to up to two previous systemic therapies, with a 24% reduction in the risk of death compared to placebo [6]. Unfortunately, the trial was not enriched as per MET expression but now cabozantinib is another treatment asset after progression under sorafenib. In 2018 Zhu *et al* reported that ramucirumab, a human IgG1 monoclonal antibody that inhibits the activation of VEGFR-2, significantly improved survival in HCC patients who progressed under sorafenib. The target population was enriched by recruiting patients with increased alpha-fetoprotein (AFP) ≥ 400 ng/mL [7] so that this is the first positive phase III study conducted in a biomarker-selected population. The mechanism underlying the benefit in high-AFP patients, while this not existing in low AFP patients is unknown. Thus, the full acceptance of AFP as a predictive factor in addition to its known prognostic value, still has to wait.

In summary, major advancements have taken place and HCC should no longer be seen as a disease with grim prognosis and marginal treatment benefits. Several agents are now proven to provide survival benefits and increased knowledge in molecular-driven mechanisms will further refine trial design and analysis so that personalized medicine becomes a reality in patients diagnosed with HCC.
